# Parliament and the Professions

**Published:** 1919-04-05

**Authors:** 


### PARLIAMENT AND THE PROFESSIONS.

### Venereal Diseases.

The Parliamentary Secretary to the Local Government Board (Major Astor) informed Major Courthorpe that the Board is not prepared, as at present advised, to propose regulations providing for the compulsory notification of venereal diseases. Mr. Churchill said he was informed that it is the practice to retain soldiers suffering from venereal disease in hospital at least as long as they are in an infective condition.

### War Charities in Scotland.

A Bill has been passed by the House of Lords and read a second time in the House of Commons which provides for funds, collected in Scotland for war charities, to be distributed in accordance with some cognate scheme which has been approved by the Local Government Board, or that a war charity shall have the right to hand over its funds to some charitable institution approved by the Local Government Board.

### National Relief Fund.

Mr. Bonar Law informed Sir K. Wood that the Government is not prepared to consider taking steps to secure Parliamentary control of the National Relief Fund.

### Inaccurate Clinical Thermometers.

Mr. Swan asked the Parliamentary Secretary to the Ministry of Munitions if the Government is prepared to suspend the Order requiring all clinical thermometers to be tested and approved by the National Physical Laboratory before sale. Mr. Kellaway replied that it was found necessary to make the Order owing to the large number of inaccurate instruments which were getting into the hands of the public, and he did not propose to suspend it. In order to develop supply arrangements are made for the manufacture of the necessary kind of glass, and the instrument makers are given expert advice in the production of the finished article. An average of 10 per cent. of the instruments submitted by chemists have been rejected.

